# Methodology for triage of urologic surgical cases in the setting of a pandemic

**DOI:** 10.1186/s12893-021-01067-9

**Published:** 2021-03-06

**Authors:** Ahmed Aboumohamed, Josh Gottlieb, Matthew DeMasi, Emily Barry, Alexander Sankin, Kara Watts

**Affiliations:** 1 Department of Urology, Montefiore Medical Center, Bronx, NY USA; 2 Albert Einstein College of Medicine, Bronx, NY USA

**Keywords:** COVID-19, Pandemic, Triage, Urology

## Abstract

**Background:**

The first wave of the COVID-19 pandemic in March 2020 forced our healthcare system in the Bronx, New York to cancel nearly all scheduled surgeries. We developed a framework for prioritizing postponed urologic surgeries that was utilized once cases were permitted to be rescheduled. As many parts of our country experience first and second waves of this pandemic, our framework may serve as a resource for other centers experiencing restrictions on the scheduling of elective urologic surgeries.

**Methods:**

As the COVID-19 pandemic started and peaked in New York, almost all of our scheduled urologic surgeries were cancelled. Each Urologist was asked to rank his/her cancelled surgeries by priority (Level 1—least urgent; Level 2—moderately urgent; Level 3—most urgent). A committee of Urologists assigned a subclass to Level 3 and 2 cases (3a—least urgent; 3b—moderately urgent; 3c—most urgent; 2a—lower priority; 2b—higher priority). The committee then reviewed cases by urgency to derive a final priority ranking.

**Results:**

A total of 478 total urologic surgeries were canceled and categorized: 250 Level 1, 130 Level 2, 98 Level 3 (73 adult, 25 pediatric). Level 3c involved renal cell carcinoma ≥ T2b, high-grade bladder urothelial carcinoma, adrenal mass/cancer > 6 cm, testicular cancer requiring radical orchiectomy, and penile cancer. Level 3b involved T2a renal masses requiring nephrectomy, while high-risk prostate cancer and symptomatic nephrolithiasis were classified as 3a. Level 2 included testicular cancer requiring retroperitoneal lymph node dissection and complicated benign prostatic hyperplasia. Surgeries for urologic reconstruction, non-complicated nephrolithiasis, erectile dysfunction, and urinary incontinence were considered Level 1.

**Conclusions:**

Our disease-specific approach to surgical rescheduling offers appropriate guidance for triaging urologic surgeries. Our system can provide guidance to other institutions as COVID-19 cases surge in different regions and with the growing second wave.

## Background

The SARS-CoV-2 (COVID-19) pandemic has had unprecedented effects on the United States healthcare system in 2020. One of the earliest and largest viral outbreaks in the United States occurred in New York City (NYC), with approximately 45,000 confirmed cases by the end of March, over 200,000 cases by the end of May, and almost 380,000 by mid-December [1]. The rapid progression of this outbreak forced healthcare systems to restructure their healthcare delivery with minimal notice. Our academic institution is located in the Bronx, NYC’s borough with the 2nd highest rate of COVID-19 cases and a reputation as the initial ‘epicenter’ of the pandemic [1]. Our institutional response, like many, was rapid and dramatic—expanding hospital bed capacity, increasing intensive care unit space and deploying medical personnel of varied specialties to the management of COVID-19 patients. These changes necessitated a cancellation of non-emergent surgical cases, putting innumerable patients on indefinite hold.

Recently, COVID-19 infections have precipitously risen throughout the United States, including resurgences in areas that were already considered “hard-hit” areas, such as New York City. There is a possibility that non-emergent surgeries will be delayed once again. These surgeries will, of course, have to be rescheduled in the future, and from our experience, the task of triaging and prioritizing these patients awaiting surgery is an enormous feat. Our method for handling our surgical rescheduling was guided by recommendations from the American College of Surgeons, which has urged organizations to establish a scoring system for the prioritization of surgical cases [2]. This priority system is particularly important in urology, as delays in care can be associated with poorer outcomes, progression of cancer, and increased morbidity and mortality. More so, surgery is considered a gold standard for many urological conditions and malignancies [3].

Several recent papers provide specific guidelines on how to triage pending surgical cases [4–10]. We developed a unique and independent triage system of guidelines, during a worldwide pandemic in one of the most affected areas, to best suit the needs of urologic patients at our institution. Our systematic approach and framework for triaging surgeries models these recommendations and is informed by specialist guidance in our department. This can potentially serve as a framework for other institutions facing a similar cancellation of elective surgeries as COVID-19 cases surge elsewhere or for those facing an unfortunate upcoming second wave.

## Methods

**Fig. 1:**
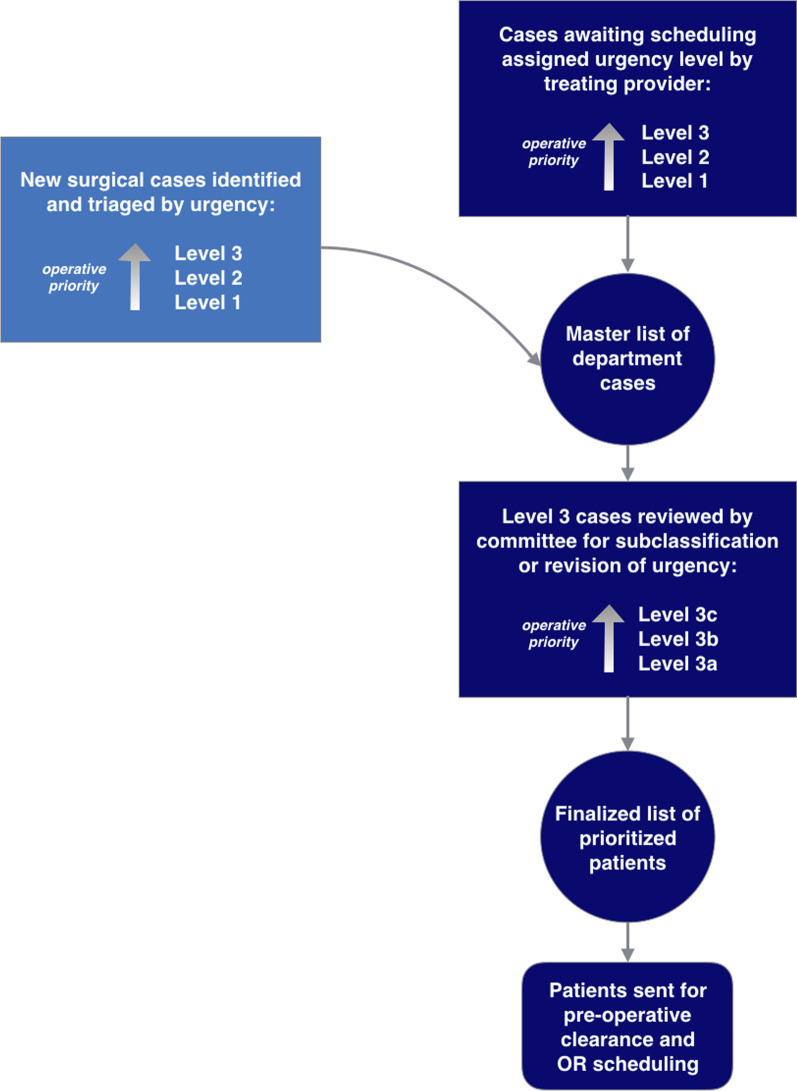
Flowchart of the prioritization of cancelled surgical cases

Our process for facilitating the rescheduling of surgeries is shown in Fig. 1 and detailed as follows: A list of all pending non-emergent urologic surgeries scheduled between March 16, 2020 and June 1, 2020 was compiled as these cases were placed on a scheduling hold. Each surgeon was provided a list of his/her individual surgical cases that were postponed due to COVID-19 and was asked to rank each case by the following priority categorization:

1. Least urgent
2. Moderately urgent
3. Most urgent

Factors that contributed to the level of urgency included, but were not limited to: date of initial diagnosis (particularly for oncologic cases), age of the patient (the younger the patient, the higher rank on the list), risk of infections, and potential impact of treatment delay on disease outcome. The patient/disease-specific information used for this priority categorization was obtained via our institution’s electronic medical record (EMR). Patients requiring emergent surgery were excluded from our master list, as emergent surgeries proceed to the operating room as promptly as possible, regardless of other pended non-emergent cases.

A committee of three adult urologists and one pediatric urologist then reviewed all individual cases submitted by each surgeon, collectively creating a department priority list. To facilitate this process, adult cases were reviewed by the three adult urologists while the pediatric cases were reviewed by the pediatric urologist on the committee. Each member reviewed all cases in his/her assigned list. The most urgent cases were reviewed first, with each reviewer assigning a subclass to Level 3 cases according to urgency (3a—least urgent; 3b—moderately urgent; 3c—most urgent) after reviewing patient-specific factors and discussing with the treating urologist if needed. This subclassification system was not necessary for pediatric cases due to the small number of urgent pediatric surgeries. To mitigate bias in the final ranking, adult committee members did not review their own cases; however, for pediatric cases, this was not feasible due to the limited number of pediatric urologists available.

The committee then reviewed all cases by their respective level of urgency and agreed on a final priority ranking for all Level 3 cases.

The above process was then completed for Level 2 cases, with subcategorization as follows: 2b—higher priority; 2a—lower priority.

It should be noted that throughout the period of restricted access to the operating room during the pandemic, each urologist was responsible for regularly reviewing his or her patients to identify potential changes in clinical status. If a patient’s clinical status deteriorated, the respective urologist submitted a request to the committee for review and potential reclassification of surgical priority ranking.

During the maximal surge of the pandemic, operating room (OR) access was reserved almost exclusively for emergency and highly urgent cases. After a period of several weeks, as the number of COVID-19 cases started to decline in the second half of May 2020, OR availability slowly increased, and cases were allowed to be scheduled based on the level of priority within our department and within our department’s allotted OR time. The most urgent cases were requested/performed first in a stepwise approach moving towards less urgent cases. This approach needed extensive coordination with OR, anesthesia, and other hospital teams to ensure available resources.

Statistical analyses performed at this time comprised simple calculations of the number of patients in each category in order to help us with projected needed OR time as OR time became more available.

## Results

Using our systematic approach, a total of 478 cases that had already been scheduled were placed on indefinite hold. Based on information submitted by our departmental faculty, we formally reviewed each case and categorized them as follows: 250 Level 1, 130 Level 2, 98 Level 3. Of the Level 3 cases, there were 73 adult cases and 25 pediatric cases.

Using urologic disease-specific guidelines, we were able to create a master table to rank different disease states and their respective surgeries according to their level of urgency (Table 1). Level 3c (18 cases, 25%) was considered the most urgent, and involved organ-confined renal cell carcinoma ≥ T2b, known or suspected high-grade bladder urothelial carcinoma (UC)/carcinoma in situ (CIS), high-grade upper tract urothelial carcinoma, adrenal mass/cancer > 6 cm, testicular cancer requiring radical orchiectomy, and penile cancer. Level 3b (12 cases, 16%) involved T2a renal masses requiring either radical or partial nephrectomy. High-risk organ-confined prostate cancer, symptomatic obstructive nephrolithiasis, and renal masses ≤ T1b were classified as 3a (43 cases, 59%).

**Table 1:** Urgency classification for triaging of urologic conditions and surgeries

Class	Subclass	Condition	Surgery	Classification rationale
3	**3c**	Bladder cancer (CIS or high-grade UC i.e. MIBC or recurrent or persistent NMIBC)	Radical cystectomy	Delays in cystectomy are adversely related to survival outcomes.
			TURBT for suspected bladder cancer	Early diagnosis and treatment are essential to optimize outcomes.
		High-grade UTUC	Nephroureterectomy	Delays in surgery are associated with adverse outcomes.
			Ureteroscopy and biopsy if suspected	Early diagnosis and prompt subsequent management is essential for optimal outcomes.
		Kidney cancer (organ confined T3+ , tumors with renal vein or IVC thrombus, tumors > 10 cm)	Radical nephrectomy +/− tumor thrombectomy	High risk of early spread and adverse outcomes.
		Testicular cancer	Radical orchiectomy	Limited survival data but concerns for early metastasis.
		Adrenal tumors; > 6 cm	Adrenalectomy	High risk for being cancerous and subsequent early spread.
		Penile cancer	Total or partial penectomy	Limited data but early surgery may prevent lymphatic spread.
	**3b**	Kidney cancer	Radical Nephrectomy for renal tumors 7–10 cm	High risk for growth and metastasis.
			Partial nephrectomy for T2 renal tumors/tumors in a solitary kidney	Large renal tumors may grow too large for a possible partial nephrectomy if delayed.
	**3a**	Prostate cancer (high-risk)	Radical prostatectomy if organ confined	Potential increased risk in BCR and adverse outcomes with delays in management.
		Kidney cancer	Radical or partial nephrectomy for T1b renal tumors	Risk of cancer extension and metastasis. Growth of tumors during a delay may prevent possible a partial nephrectomy.
		Obstructing symptomatic nephrolithiasis; non-stented or with stent > 6 months	Ureteroscopy PCNL	Risk of permanent renal damage with long-term obstruction, pyelonephritis, and severe encrustation with stents > 6 months.
2	**2b**	Bladder cancer (low-grade UC)	TURBT for bladder recurrences of known low grade UC	Low-grade UC is slow growing with non-aggressive features. Delays can be several months without potentially affecting outcomes.
		PCa (unfavorable, intermediate risk)	Radical prostatectomy	Potential increased risk in BCR and adverse outcomes with delays in management.
		Testicular cancer	Primary or post-chemotherapy RPLND	Limited data regarding the effect of delayed surgery.
	**2a**	Kidney cancer	Partial nephrectomy for T1a renal tumors	Low risk of progression and metastasis.
		PCa (favorable, intermediate risk)	Radical prostatectomy	Low risk of spread and progression.
		Complicated BPH	TURP Simple prostatectomy	Risk of the condition worsening with severe infection or bleeding.
1		Miscellaneous Urethral stricture Incontinence req. AUS Erectile dysfunction	Reconstructive surgery Urethroplasty Ureteral re-implant AUS IPP	Condition can be stabilized by drainage (Foley, SPT, or PCN); quality of life conditions with no medical urgency.
		Female urology, incontinence Pelvic prolapse	Urethral diverticulectomy Sacrocolpopexy Prolapse repair, etc.	Quality of life conditions with no medical urgency.
		Infertility	Microsurgical Recon. Sperm extraction	
		Non-complicated BPH	TURP Simple prostatectomy	
		Non-complicated nephrolithiasis	Ureteroscopy/PCNL	

*AUS* artificial urinary sphincter, *BCR* biochemical recurrence, *BPH* benign prostatic hyperplasia, *IPP* inflatable penile prosthesis, *MIBC* muscle-invasive bladder cancer, *NMIBC* non muscle-invasive bladder cancer, *PCN* percutaneous nephrostomy, *PCNL* percutaneous nephrolithotomy, *PCa* prostate cancer, *RPLND* retroperitoneal lymph node dissection, *SPT* suprapubic tube, *TURBT* transurethral resection of bladder tumor, *TURP* transurethral resection of prostate, *UC* urothelial carcinoma, *UTI* urinary tract infection, *UTUC* upper tract urothelial carcinoma

Level 2 cases included testicular cancer requiring retroperitoneal lymph node dissection, and complicated benign prostatic hyperplasia (BPH) cases requiring transurethral resection of prostate or simple prostatectomy. Surgeries for urologic reconstruction, infertility, erectile dysfunction, incontinence, pelvic organ prolapse, uncomplicated BPH, and mildly or asymptomatic nephrolithiasis were considered Level 1.

As the pandemic is still ongoing and follow up time is limited, we have not yet assessed patient/disease-specific outcomes as a result of surgical prioritization to evaluate the efficacy of this methodology.

## Discussion

As the number of hospitalizations for COVID-19 in our heavily-hit region started to decline toward the end of the first wave, we were faced with the challenge of triaging and rescheduling surgeries that were postponed due to the pandemic. In order to approach that challenge in a way that most equitably served our patients, specialty, and institution, we developed a systematic approach for surgical prioritization. Our approach implicitly favored patients with more time-sensitive diagnoses, wherein a delay in surgery may alter their outcome. Consideration was also given to other factors, such as the date of initial diagnosis (particularly for oncologic cases) and the age of the patient (the younger the patient, the higher rank on the list).

Several other statements have offered guidance on how to approach prioritization of surgeries in light of limited hospital resources or operating room availability. Wallis et al. formulated a collaborative review of the risks associated with delayed treatment of urological cancers. Based on this review, patients with high-grade urothelial carcinoma, advanced kidney cancer, testicular cancer, and penile cancer should be prioritized as more urgent [4]. Stensland et al. developed additional suggestions. Specifically, acute infections (i.e., abscesses and Fournier’s gangrene) and ischemic or traumatic conditions are considered urgent procedures warranting priority. In our proposed methodology, these particular cases were excluded from the triage process since they are deemed emergent/highly urgent and surgical interventions were typically permitted on a case-by-case basis. Surgeries to correct benign prostatic hyperplasia, incontinence, and infertility are elective, and therefore less urgent [5].

Quaedackers et al. additionally described suggestions for postponed pediatric urologic surgeries. Similar to adults, benign scrotal and penile surgeries, as well as surgery for incontinence, uncomplicated urolithiasis, and vesicoureteral reflux can be safely delayed. Other conditions that may cause irreversible progression of disease, organ damage, or are life-threatening should be prioritized to continue. These include surgeries to correct complicated obstructions, testicular torsion, and oncological malignancies [6].

These sources were influential in developing our ranking system, yet they lacked a guideline model for developing a prioritization list. Thus, our model develops an urgency prioritization system, largely based on these prior studies, while also giving consideration to time-sensitive diagnoses during which a delay would alter outcome. This prioritization guideline was necessary as our location in the Bronx cancelled all nonemergent surgeries—thus, our backlog of surgical cases consisted of numerous critical and oncological, time-sensitive cases.

Another model developed a similar triaging system that focused on the potential harms that would result from delaying surgery [11]. They assigned procedures to five tiers, with Tier Zero cases requiring emergency surgery and Tier Four cases consisting of nonessential procedures. Although we had a similar approach to triaging patients, our department, actually had to cancel all non-emergent surgeries as a result of the severe strain COVID-19 cases had on our hospital system. Thus, we had to prioritize a large backlog of patients awaiting surgeries that were not purely nonessential, and we have a system that was amply tested with the task of incorporating patients back into surgical practice.

Prachand et al. developed the MeNTS system, which assigns a numerical score to each patient for overall surgical prioritization [7]. Scores are calculated by a number of variables, including but not limited to patient demographics, status and urgency of disease or diagnosis, and hospital and surgical resources required. Unlike previously referenced systems, surgeries requiring higher resource allocation will lose points in prioritization.

In contrast to MeNTS, our system is based on disease status and prognosis as a surrogate for surgical urgency. If hospital resources are a contributing variable, patients requiring more complex surgery, or who are more medically comorbid and at a high risk for surgical complication, may get penalized on the priority ranking for this. While hospital resources are very important to consider, particularly when it comes to the ability to care for a patient during and after surgery, we fear this may lead to delays in surgery in select patients who require prompt intervention. Except for the first several weeks of the pandemic when hospital resources were markedly restricted and not adequately prepared to deal with the immense pandemic, we do not feel a current significant need for including hospital resources as a major determinate in triaging surgical patients.

Our system has an advantage over guidelines that assign numeric scores for varying categories. Such systems have a potential misconception that all variables have equal numerical value. As acknowledged in the MeNTS article, not every aspect of a patient’s disease, procedure, or demand on the hospital system is quantitatively proportionate. While numerical scoring can still prove to be quite useful, it may give a false sense of objectivity due to significant subjectivity involved in assigning several of these numerical scores. Assigning class and subclass allows us to triage cases using disease status and rationale for surgical urgency, without being skewed by the numeric values of many other variables that may not be of equal significance.

Another advantage to our approach is that these guidelines can remain relevant even after the COVID-19 pandemic has passed. Utilizing surgical guidelines that consider patient’s varying pathologies, disease status, as well as potential outcomes from a delay in surgery is extremely useful for surgical planning regardless of the current pandemic and resource limitations.

Our guideline system has some limitations to acknowledge. We based our system on the impact of delayed treatment on the diagnosis in question but did not consider the burden on resources necessary for particular surgical procedures or high-risk surgical candidates. At a time when ventilators and ICU beds are extremely limited, this could be a legitimate roadblock to performing complex surgeries on higher acuity patients despite the potentially aggressive nature of their disease. As discussed by Puliatti et al., cancer patients that we would consider Level 3 cases are at especially high-risk group for COVID-19 complications [12]. If these patients are admitted to the hospital for a urologic surgery and are exposed to COVID-19, their disease course might require another hospitalization and further consumption of limited resources. An alternative approach would be to encourage the use of chemotherapy and radiation on an outpatient basis; this approach considers the patient’s overall survival at a time when hospitalizations pose increased risks to patients [13]. However, our system has the advantage in that it will allow adjustments to top-priority patients at any time to address the current state of hospital resource availability.

Although our rationales for determining surgical urgency are rooted in evidence-based knowledge and current standard practice, we faced another limitation in the lack of definitive data regarding the impact of delayed intervention on survival outcomes. For example, there are limited data on the impact of delayed radical orchiectomy on survival for testicular cancer. Due to logical concerns of metastasis and disease progression, it is still common practice to perform radical orchiectomy as early as possible despite a lack of solid survival data. Thus, some rationales are based on informed specialist opinion and common practice, which may decrease objectivity.

Lastly, it is important to acknowledge that as the pandemic is still ongoing and follow up time is limited, we have not yet assessed patient/disease-specific outcomes as a result of surgical prioritization to evaluate safety and efficiency of this methodology. However, we do hope to assess patient/disease-specific outcomes as a result of our triage methodology in the future.

## Conclusion

As happened at our institution in the beginning of the pandemic, there are ongoing concerns that surgical cases may again be delayed during this ongoing second wave. Overall, we believe our disease-specific approach to surgical rescheduling offers appropriate guidance to other institutions as cases begin to surge in different regions. We believe our already tested framework for which we triaged our patients to be rescheduled after the initial COVID-19 pandemic, in the epicenter of the country, could be a useful model for those who might experience similar unfortunate circumstances. While patient factors and hospital resources can lead to adjustments in the initial ranking, we do believe this simplified system can be valuable in determining the initial triage list.

## Data Availability

Not applicable.

## Abbreviations

SARS-CoV-2: COVID-19
NYC: New York City
EMR: Electronic medical record
OR: Operating room
UC: Urothelial carcinoma
CIS: Carcinoma in situ
BPH: Benign prostatic hyperplasia
